# Associations between local rates of violence and experiences of psychosis in Trinidad

**DOI:** 10.3389/fpubh.2025.1570957

**Published:** 2025-06-11

**Authors:** Tessa Roberts, Joni Lee Pow, Casswina Donald, Diego Quattrone, Gerard Hutchinson, Craig Morgan

**Affiliations:** ^1^Social Genetic and Developmental Psychiatry Centre, Institute of Psychiatry, Psychology & Neuroscience, King’s College London, London, United Kingdom; ^2^Department of Biomedicine, Neuroscience and Advanced Diagnostics, Psychiatry Section, University of Palermo, Palermo, Italy; ^3^Department of Psychiatry, The University of the West Indies, St. Augustine, Trinidad and Tobago

**Keywords:** psychosis, place, violence, Trinidad, Caribbean

## Abstract

**Introduction and aim:**

Individual-level exposure to violence is known to influence various aspects of the experience of psychosis. This study aimed to assess the impact of local violence rates on the symptom profiles and outcomes of people with psychosis, their exposure to trauma and other potential risk and protective factors, and interactions with the police and mental health services, in a Caribbean country with high rates of violent crime.

**Methods:**

Data from 212 people with psychosis and matched population control participants were collected through a population-based programme of research on psychosis in Trinidad (INTREPID II) and linked geographically with crime statistics, disaggregated to the areas surrounding each police station.

**Results:**

There was no evidence of a substantive association between local rates of violent crime and symptoms of psychosis or the course of illness on most measures, although people in lower crime areas appeared to be more likely to experience hallucinations than those living in high-violence areas (β-0.30, 95% CI −0.50–−0.11, *p* < 0.01). There was some evidence that people living in high-violence areas were more likely to be restrained within the mental health system than those from lower-violence areas (OR 2.00, 95% CI 0.98–4.09, *p* = 0.06), despite being no more likely to act in violent or disruptive ways.

**Conclusion:**

The lack of association between violent crime and various aspects of psychosis may indicate that the psychological effect of violent crime is not confined to single localities, and may be influenced by other factors besides rates of violence in participants’ immediate local area. Research is needed to investigate why individuals from disadvantaged neighbourhoods that are associated with gang violence experience different treatment within health services and to evaluate strategies to address these disparities.

## 1 Introduction

At the individual level, there are clear links between experiences of violence and psychosis. Multiple lines of evidence suggest that exposure to childhood abuse—particularly violence, either physical or sexual—shapes experiences of psychosis, including risk of psychotic experiences (1), symptom profiles (2, 3), symptom severity (4), persistence of psychotic experiences and disorder (5), interactions with the mental health system (6), and the course of illness (7). Risk of psychosis is further exacerbated among those who experienced abuse during childhood when they are also exposed to abuse or violence in adulthood (2, 8), and exposure to stressful events in adulthood is associated with relapse (9, 10). Some evidence suggests that increased threat anticipation in the face of stressors, among those exposed to childhood adversity, might underlie these observations (11), while other models implicate affective pathways (via anxiety/depression) (12) or dissociation (13, 14) as key mechanisms linking exposure to violence and other forms of trauma to psychosis.

There is also some evidence that rates of violence at the level of neighbourhoods or local areas may be associated with variation in some aspects of psychosis experiences. Differences have been reported in risk of psychosis between local areas (15), with an approximately 2.4-fold greater risk in urban than rural areas (16), at least within Northern Europe. In UK studies, crime victimisation has been implicated in UK studies as an important factor that may explain higher rates of psychotic experiences in urban areas (17–19). However, the evidence base is limited in that place effects in psychosis have almost exclusively been investigated in Europe and North America. A large cross-sectional study of psychotic experiences in 35 low- and middle-income countries found an association between criminal victimisation and sub-clinical psychotic symptoms (among the general population) at the individual level (20), but whether this implies differences in clinically diagnosable experiences between areas with higher or lower levels of violent crime is unclear. The relationship between living in neighbourhoods afflicted by violence and experiences of psychosis warrants more detailed investigation across a range of contexts to better understand the impact of living in such areas.

In Trinidad, where reported rates of psychosis are substantially higher than global pooled estimates (21), rates of violent crime have risen substantially over the past two to three decades, and Trinidad & Tobago now has one of the highest homicide rates in the world (22, 23). This has been linked to changes in transnational narcotics trade routes, which has brought increased gang violence to the island, and an influx of firearms that has increased the lethality of violence between rival gangs (24, 25). Although violence is now endemic across the country, it is especially concentrated within deprived urban areas where young people—particularly young men and boys—are most susceptible to recruitment by criminal gxangs. In the INTREPID II programme (a 5 year epidemiological study of psychosis in India, Nigeria and Trinidad (26)) we found major differences in rates of psychosis between local areas, with much higher rates in the urban areas that have been most affected by gang violence (27).

Understanding whether local levels of violence are associated with different experiences of psychosis is important for planning services and prevention strategies, and allocating public health resources appropriately. Qualitative research with people with a diagnosis of psychotic disorder in Trinidad implicated violent crime within one’s neighbourhood as an important factor affecting their wellbeing and recovery (28). We hypothesise that in areas characterised by high rates of violent crime, we will see a distinct profile of needs among people with psychosis and a more negative course of illness. This study aims to explore the relationship between living in an area affected by high rates of violent crime and experiences of psychosis.

### 1.1 Aims and objectives

This study takes an exploratory, hypothesis-driven approach to explore the association of neighbourhood violence with various dimensions of psychosis.

#### 1.1.1 Objectives

This exploratory study tested the following hypotheses:

1. People living in high-violence areas—both those with and without psychosis—will report greater exposure to violence than those living in lower-violence areas;
2. People with psychosis living in high-violence areas will experience more positive symptoms and paranoia compared to those living in lower-violence areas;
3. People with psychosis living in high-violence areas will be more likely to have contact with the police and to be involuntarily admitted to hospital than those living in lower-violence areas;
4. People with psychosis living in high-violence areas will be less likely to have a favourable clinical course of psychosis, and have lower levels of social functioning at follow-up than those living in lower-violence areas.

## 2 Methods

### 2.1 Participants and recruitment

INTREPID II is a multi-site research programme in India, Nigeria and Trinidad to investigate the epidemiology of psychotic disorders across diverse settings (26). As part of this programme, a team of researchers at the University of the West Indies in Trinidad identified all those who presented to public mental health services within the catchment area (comprising Diego Martin, Port of Spain, San Juan/Laventille, Tunapuna/Piarco, Arima, Chaguanas, and Sangre Grande) who met criteria for a diagnosis of psychotic disorder and had not previously received 1 month or more of continuous treatment with antipsychotic medication. These individuals were subsequently invited to participate in a case–control study as part of the INTREPID II programme. 212 individuals were recruited and interviewed between 2018 and 2020, and were subsequently followed up for approximately 2 years. All participants had a diagnosis of psychotic disorder (with no identifiable organic cause) confirmed by a local psychiatrist following a diagnostic interview, were aged 18–64 at the time of recruitment into the INTREPID II programme, had not been treated with antipsychotics for more than one continuous month at the time of identification, and were resident within the catchment area.

### 2.2 Setting

The dual-island nation of Trinidad and Tobago is the southernmost country in the Caribbean, with a population of around 1.37 million. The catchment area for INTREPID II includes seven municipalities with an ethnically diverse population (40% Afro-Trinidadian, 30% Indo-Trinidadian, 20% mixed heritage, plus small Caucasian, Syrian/Lebanese and Chinese minority populations) and substantial economic inequality. It includes both urban and rural areas.

### 2.3 Data collection and measures

#### 2.3.1 Primary exposure variable

Neighbourhood violent crime levels were calculated using publicly available crime statistics from the Central Statistical Office of Trinidad & Tobago,^1^ reported by police station. We used 2018 statistics, which corresponds to the first year of the INTREPID II baseline data collection. We grouped crime data into violent and non-violent crimes in order to estimate rates of violent crime in each local area. For the purposes of the current analyses, we included the following pre-specified categories in our estimates of violent crime: murder, assault, rape, sexual assault and other sexual offences, kidnapping, burglary, robbery, possession of firearms and ammunition.

**Figure 1:**
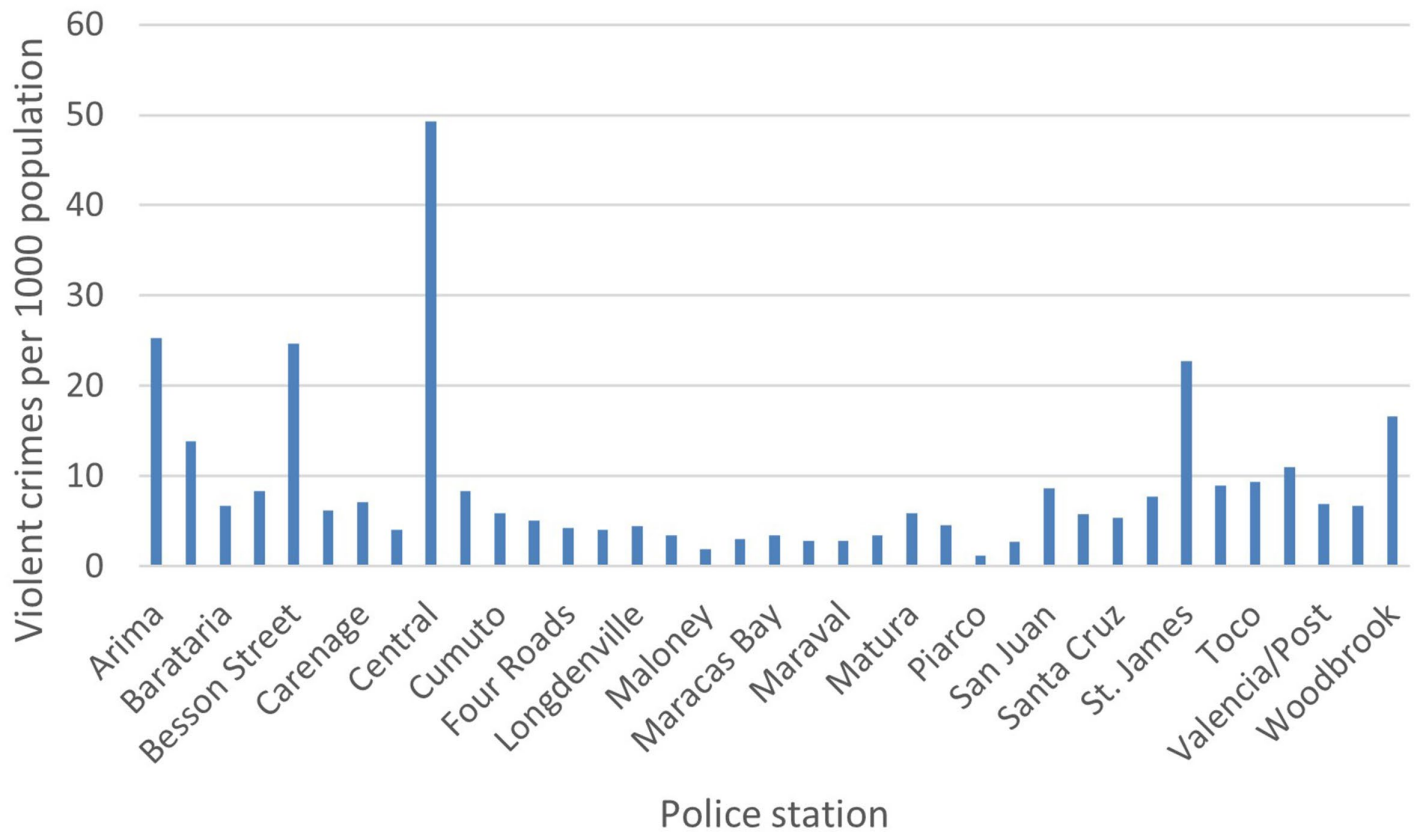
Rate of violent crime by police station.

We used ArcGIS (29) to estimate catchment areas for each police station by constructing Thiessen polygons around each station, indicating the area within which this station was nearer than any other. We calculated the underlying population for these catchment areas using 2020 data (the closest available to the 2018 crime statistics) from the Global Human Settlement Layer 2022 data package (30, 31). This is a raster dataset of population distribution estimates that combines various sources including disaggregated census data, UN projections and density of built-up areas using satellite imagery, using the Zonal Statistics tool from the Spatial Analyst package in ArcGIS. We used these data to standardise crime rates relative to the size of the local population. After plotting the distribution of violent crime by local area (defined as the area around each policy station) we divided these into high-violence and lower-violence areas, with more or less than 10 violent crimes within a 1 year reporting period per 100,000 population, since the data appeared to have a bimodal distribution (see Figure 1).

#### 2.3.2 Data linkage

Individual data from the INTREPID II study was linked using the Link tool in ArcGIS with the police station catchment area data using GPS coordinates that were collected by researchers at the time of the baseline INTREPID II interviews, using a mobile phone. GPS coordinates were cross-checked against participants’ recorded addresses prior to data linkage.

#### 2.3.3 Outcome variables

Potential participants were first screened using the Screening Schedule for Psychosis (32), and diagnoses were then confirmed using the Schedules for Clinical Assessment in Neuropsychiatry (SCAN) (33). Demographic data were collected using the MRC Sociodemographic Schedule. The same researchers also collected data using the Personal and Psychiatric History Schedule (32), the Global Assessment of Functioning (34), the Harvard Trauma Questionnaire (35), the Child Trauma Questionnaire (36), and the Alcohol, Smoking and Substance Involvement Screening Test (37). SCAN interviews were conducted by trained researchers and then reviewed by a psychiatrist to assign diagnoses. Dimensional symptom scores were generated by converting individual variables into the OPerational CRITeria (OPCRIT) system (38) then using factor analysis to group symptom variables, resulting in one general factor and six specific factor scores. This analysis will be reported in more detail elsewhere (39), but to summarise, we generated symptom dimension scores as follows: Using OPCRIT data, we first estimated a bifactor model comprising a general symptom dimension and specific symptom dimensions using the Weighted Least Squares with Mean and Variance Adjustment (WLSMV) estimator for bifactor item response modelling in Mplus version 7.4. In our analyses, we found that general and specific symptom dimensions accounted for the majority of the variance in the dataset (McDonald’s ω: 0.94) and that model fit statistics were good and reliability indices were strong across all dimensions. We then generated general and specific factor scores using the ‘FSCORES’ function in Mplus.

### 2.4 Ethics

This study was approved by King’s College London (Reference: HR-17/18–5,601), London School of Hygiene and Tropical Medicine (Reference: 15807), the University of the West Indies (St Augustine campus) in Trinidad (Reference: CEC483/03/18), and the Eastern (Reference: PHO: 24/1), North Central (Reference: 185–43 CD), and North West (approved on 9 July 2018, no reference number issued) Regional Health Authorities in Trinidad.

### 2.5 Analysis

Data from the INTREPID II study were linked with local crime data using the GPS coordinates for participants address at the time of recruitment, using ArcGIS version 10 (29). We used logistic regression to compare the odds of living in a high-violence or lower-violence neighbourhood by the various categorical outcomes of interest, and linear regression for continuous outcomes, controlling for age, gender, ethnicity, and cannabis use, using STATA version 14 (40). To handle missing data, we used multiple imputation by chained equations (41, 42). The imputation models included all variables in the main analyses. Post-imputation analyses combined estimates across 25 imputed data sets using Rubin’s rule (43). Proportions of missingness for each variable that was imputed and sensitivity analyses are included in the Supplementary material.

## 3 Results

Table 1 summarises the characteristics of study participants. They included residents of all seven municipalities within the catchment area. Nearly half of participants (46.2% of people with psychosis and 46.7% of control participants) were aged 18–29. Just over half were male (57.6%). Participants were predominantly Afro-Trinidadian (52.8% of those with psychosis and 52.4% of control participants), with smaller proportions of Indo-Trinidadian ethnicity and of mixed heritage. Nearly two thirds of those with psychosis had a non-affective diagnosis (59.4%).

**Table 1:** Participant characteristics.

Variable	Cases (%)	Controls (%)
Municipality		
Port of Spain	29 (14.0)	22 (11.7)
Diego Martin	21 (10.1)	19 (10.1)
San Juan/Laventille	49 (23.7)	46 (24.5)
Tunapuna/Piarco	43 (20.8)	38 (20.2)
Arima	13 (6.3)	13 (6.9)
Chaguanas	24 (11.6)	24 (12.8)
Sangre Grande	27 (13.0)	26 (13.8)
Age group		
18–29	98 (46.2)	99 (46.7)
30–39	61 (28.8)	66 (31.1)
≥40	53 (25.0)	47 (22.2)
Gender		
Male	122 (57.6)	122 (57.6)
Female	90 (42.5)	90 (42.5)
Ethnicity		
Afro-Trinidadian	112 (52.8)	111 (52.4)
Indo-Trinidadian	41 (19.3)	45 (21.2)
Mixed	59 (27.8)	54 (25.5)
Other	0 (0)	2 (0.9)
Diagnosis		
Affective	81 (38.2)	N/A
Non-affective	126 (59.4)	N/A
Substance-induced	5 (2.4)	N/A

**Figure 2:**
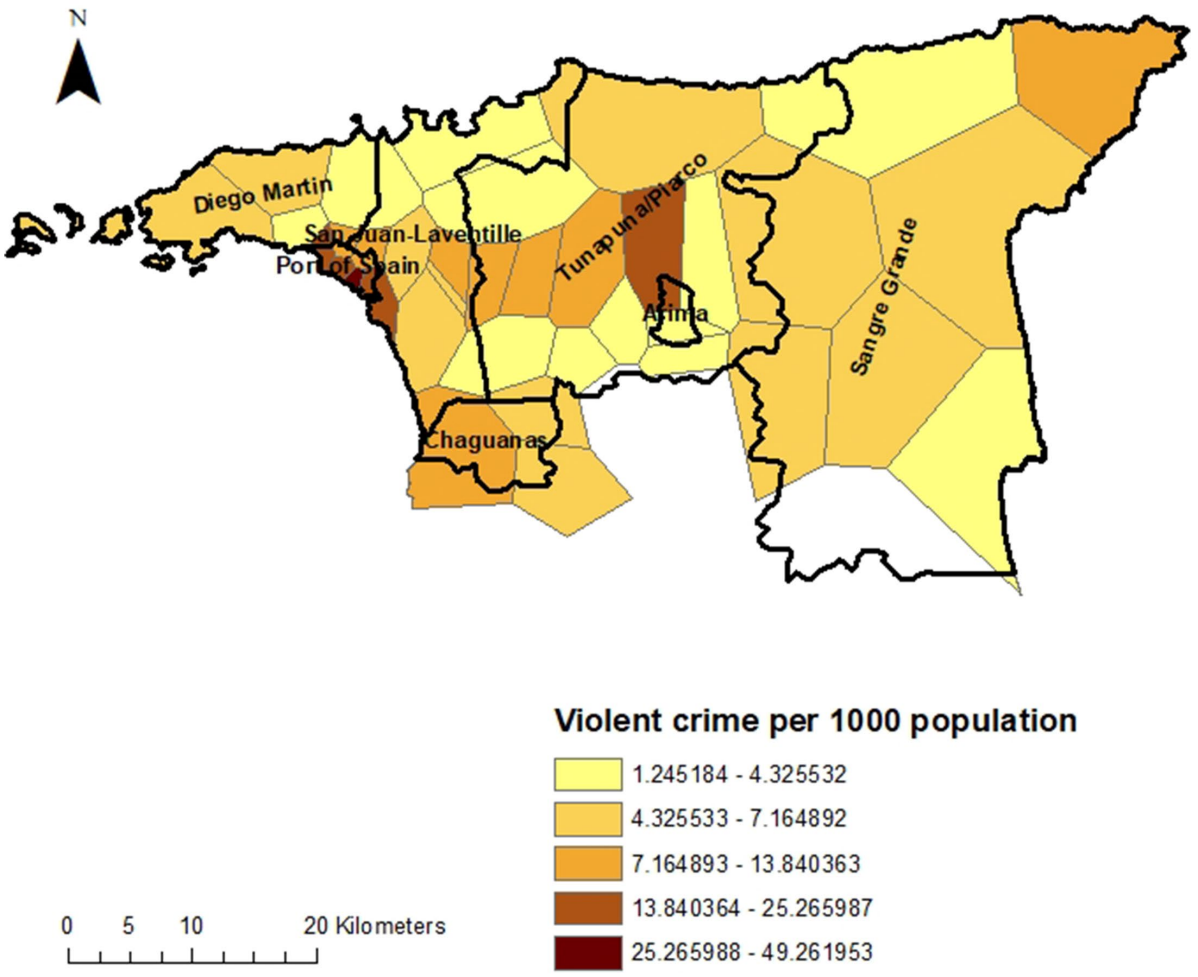
Rate of violent crime by local area.

Figures 1 and 2 show the rate of violent crime per 1,000 population across the catchment area of INTREPID II. The rates are highest in the urban areas, particularly around the capital, Port of Spain, and Arima, with variation by local area within these municipalities.

Table 2 shows the prevalence of potential risk and protective factors in high- and lower-violence areas in the first round of data collection for INTREPID II, among all participants (those with psychosis and control participants). There was no substantive difference in the prevalence of frequent or problematic cannabis use, directly experiencing or witnessing traumatic events, living alone or identifying as Afro-Trinidadian.

**Table 2:** Baseline risk/protective factors and their association with neighbourhood violence (cases and controls).

Variable	Lower-violence neighbourhoods (<10 per 100,000 population), *n* (%)	High-violence neighbourhoods (>10 per 100,000 population), *n* (%)	Unadjusted odds ratio (95% confidence interval)	*p*-value
Problematic cannabis use (ASSIST score>4)	98 (33.5)	42 (35.9)	1.09 (0.67–1.77)	0.73
Frequent cannabis use (weekly or more)	67 (22.9)	31 (26.5)	1.16 (0.69–1.95)	0.58
% experienced any traumatic event (HTQ)	264 (69.1)	112 (64.7)	1.61 (0.78–3.35)	0.20
% witnessed any traumatic event (HTQ)	116 (38.4)	54 (44.3)	1.11 (0.83–1.95)	0.27
Living alone	37 (12.3)	20 (16.5)	1.34 (0.70–2.56)	0.38
Afro-Trinidadian ethnicity	152 (50.3)	71 (58.2)	1.37 (0.90–2.10)	0.14

Tables 3 and 4 show how the symptom profiles of those with psychosis varied between high- and lower-violence areas in the first round of data collection for INTREPID II. There was little evidence of a difference in symptoms between those living in high-violence and lower-violence areas on most measures. There was weak evidence that delusions of persecution—and to a slightly lesser extent delusions of reference—are slightly less common in high-violence than lower-violence areas. Hallucinations also appeared to be less frequent in high-compared to lower-violence areas (adjusted coefficient −0.30, *p* < 0.01).

**Table 3:** Baseline presentation and neighbourhood violence (cases only—categorical variables).

Variable	Lower-violence neighbourhoods (<10 per 100,000 population), *n* (%)/mean (95% CI)	High-violence neighbourhoods (>10 per 100,000 population), *n* (%)/mean (95% CI)	Adjusted odds ratio ^*^(95% confidence interval)	*p*-value
Affective diagnosis (F30.2, F31.2, F31.5, F32.3, F33.3)	63 (40.9)	18 (31.0)	1.51 (0.74–3.10)	0.26
Delusions of persecution (SCAN>1)	93 (61.1)	28 (48.3)	0.52 (0.28–0.99)	0.05
Delusions of reference (SCAN>1)	45 (29.8)	17 (29.8)	0.57 (0.31 1.08)	0.09

* Controlling for age, gender, ethnicity, and cannabis use.

**Table 4:** Baseline presentation and neighbourhood violence (cases only—continuous variables).

Variable	Lower-violence neighbourhoods (<10 per 100,000 population), mean (95% CI)	High-violence neighbourhoods (>10 per 100,000 population), mean (95% CI)	Adjusted beta coefficient^*^ (95% confidence interval)	*p*-value
Symptom dimensions: General factor	0.56 (0.45–0.66)	0.50 (0.33–0.68)	−0.03 (−0.23–0.17)	0.76
Symptom dimensions: Delusions factor	0.12 (0.01–0.23)	0.05 (−0.13–0.23)	−0.08 (0.29–0.13)	0.43
Symptom dimensions: Hallucinations factor	0.04 (−0.06–0.13)	−0.27 (0.45––0.09)	−0.30 (−0.50 –−0.11)	<0.01
Symptom dimensions: Negative factor	−0.04 (−0.15–0.07)	−0.04 (−0.22–0.13)	−0.02 (−0.22–0.19)	0.86
Symptom dimensions: Disorganised factor	−0.07 (−0.14–0.00)	−0.07 (−0.19–0.06)	−0.02 (−0.15–0.12)	0.81
Symptom dimensions: Manic factor	0.28 (0.18–0.38)	0.25 (0.08–0.41)	−0.02 (−0.22–0.17)	0.80
Symptom dimensions: Depressive factor	0.51 (0.39–0.63)	0.36 (0.13–0.58)	−0.09 (−0.32–0.14)	0.46
Overall symptom severity at baseline (GAF 1)	62.1 (59.8–64.4)	61.5 (58.1–65.0)	−0.40 (−6.49–5.69)	0.90
Overall disability at baseline (GAF 2)	60.9 (58.8–63.0)	59.3 (56.0–62.3)	−1.87 (−7.63–3.62)	0.50

* Controlling for age, gender, ethnicity, and cannabis use.

Table 5 describes the ways in which people with psychosis came into contact with mental health services, in high- and lower-violence areas. There was weak evidence that contact for those living in high-violence areas was less likely to have been prompted by violent or disruptive behaviour, particularly physical assault, committed by 41.6% of those in lower-violence areas compared with 29.3% in high-violence areas (adjusted OR 0.55, *p* = 0.09). People with psychosis living in high- and lower-violence areas were equally likely to be arrested or to have had their first contact for psychosis with the police, rather than health services. However there was some evidence that they were more likely to be restrained within health services than those from lower-violence areas (31.0% compared with 17.1%, adjusted OR 2.00, *p* = 0.06).

**Table 5:** Contact with mental health and justice systems and neighbourhood violence (cases only).

Variable	Lower-violence neighbourhoods (<10 per 100,000 population), *n* (%)	High-violence neighbourhoods (>10 per 100,000 population), *n* (%)	Adjusted odds ratio^*^ (95% confidence interval)	*p*-value
Committed violent or hazardous act	47 (30.5)	14 (24.1)	0.76 (0.37–1.58)	0.47
Behaviour seen as threatening or grossly annoying	78 (50.6)	24 (41.4)	0.68 (0.36–1.30)	0.25
Assaulted someone physically	64 (41.6)	17 (29.3)	0.55 (0.27–1.10)	0.09
Caused damage to property	61 (39.6)	18 (31.0)	0.61 (0.31–1.20)	0.15
Felt they were being harmed or persecuted	111 (72.1)	40 (69.0)	0.92 (0.47–1.80)	0.81
Arrested	43 (27.9)	17 (29.3)	0.82 (0.39–1.72)	0.60
First contact for psychosis was police	15 (9.9)	6 (10.7)	0.87 (0.31–2.49)	0.80
Admitted to hospital	97 (63.0)	35 (58.6)	0.80 (0.42–1.53)	0.50
Involuntary admission	72 (46.8)	29 (50.0)	1.15 (0.61–2.15)	0.67
Ever restrained in services	26 (17.1)	18 (31.0)	2.00 (0.98–4.09)	0.06

* Controlling for age, gender, ethnicity, and cannabis use.

Tables 6 and 7 summarise differences in the course of illness—among people with psychosis—between high-violence and lower-violence areas. There were minimal differences between the two groups in the likelihood of remission, of having a continuous course of illness, of being in an episode at 2 follow-up, and in symptom and disability scores at 2 year follow-up.

**Table 6:** Course/outcomes and neighbourhood violence (cases only—categorical variables).

Variable	Lower-violence neighbourhoods (<10 per 100,000 population), n (%)	High-violence neighbourhoods (>10 per 100,000 population), n (%)	Adjusted odds ratio^*^ (95% confidence interval)	*p*-value
Any remission	128 (91.4)	39 (88.6)	0.50 (0.18–1.42)	0.20
No longer receiving treatment	82 (57.8)	28 (57.1)	0.92 (0.46–1.83)	0.81
Continuous course	23 (16.3)	7 (15.6)	1.25 (0.48–3.23)	0.65
In episode at follow-up	40 (27.6)	17 (33.3)	1.33 (0.67–2.63)	0.41

* Controlling for age, gender, ethnicity, and cannabis use.

**Table 7:** Course/outcomes and neighbourhood violence (cases only—continuous variables).

Variable	Lower-violence neighbourhoods (<10 per 100,000 population), mean (95% confidence interval)	High-violence neighbourhoods (>10 per 100,000 population), mean (95% confidence interval)	Adjusted beta coefficient^*^ (95% confidence interval)	*p*-value
Overall symptom severity at 2 year follow-up (GAF 1)	58.9 (55.3–62.5)	56.3 (50.2–62.4)	−1.97 (−8.77–4.82)	0.57
Overall disability at 2 year follow-up (GAF 2)	58.3 (54.7–62.0)	56.7 (50.2–63.2)	−1.38 (−8.23–5.48)	0.69

* Controlling for age, gender, ethnicity, and cannabis use.

## 4 Discussion

### 4.1 Principal findings

We found very little evidence of any substantive differences between people with psychosis living in high-violence and lower-violence areas, in terms of overall symptom levels, course and outcomes of psychosis, prevalence of common risk factors such as cannabis use, or exposure to individual-level violence and trauma. There was weak evidence that delusions of persecution and delusions of reference may be less prevalent in high-violence than lower-violence areas, and hallucinations were less frequent among those living in high-violence areas, in contrast to our initial hypotheses. The likelihood of police involvement and involuntary admission was similar for people from all areas, but we found tentative evidence that people from high-violence neighbourhoods may be more likely to be restrained once in services, despite being no more likely to act violently.

### 4.2 Strengths and limitations

This study has several strengths as well as limitations that are important to note. Very few epidemiological studies of psychosis have been conducted in the Caribbean, and none to date have examined the association between experiences of psychosis and neighbourhood-level factors such as rates of violent crime, which is currently a major public health issue in this region. Worldwide, studies of how neighbourhood-level factors affect mental health—particularly severe mental illness—remain scarce (44), limiting our understanding of how processes at the neighbourhood level shape population mental health. This study uses a population-based sample of people with psychosis, in contrast with most studies of psychosis that rely on clinical samples, which increases the representativeness of the data. Over 200 participants also represents a fairly large sample, in the context of population-based studies of a rare condition (45).

The main limitations that should be acknowledged when interpreting these findings relate to the measure of our key exposure of interest; rates of violent crime. Uncertainty in these estimates arises from various sources. Firstly, crime statistics are based on police reports, but not all crimes are likely to be reported to police. In neighbourhoods where trust in the police is lower (for instance, because police officers in the area are known to have links to gangs or are suspected to be corrupt) there may be lower rates of reporting than in neighbourhoods where the community have more positive relations with the police. In addition, some crimes may be particularly under-reported, such as domestic violence. The lack of variation that we found between high-violence and lower-violence areas in terms of individual experiences of trauma may be an indication that the officially reported crime rates may not correspond closely to actual experiences of violence. Furthermore, we have assumed that victims of crime typically report the crime to their nearest police station, which may not always be the case. This introduces some uncertainty in our measure of the exposure of interest, and we cannot rule out the possibility of systematic bias in this measure between neighbourhoods. Further uncertainty is introduced because the most recent available crime statistics were collected in 2018, whereas the population data used to standardise the data by local population are from 2020. Finally, participants’ residence was based on their address when baseline data for INTREPID were collected (between 2018 and 2020). It was not possible to account for their migration history or migration after this point. It is therefore possible that some of the null findings are attributable to error in measuring neighbourhood rates of violence.

It is also important to note that the data on course and outcomes for psychosis were taken from the 2 year follow-up of the INTREPID II study. Although this study had low attrition (185 of 212 were successfully followed up, giving an 87% retention rate), low to follow-up was greater in high-violence than low-violence neighbourhoods (22% versus 9%) which may have skewed the results on psychosis trajectories. It is also possible that some symptoms and experiences were systematically more under-reported by those living in high-violence areas, either because they have become accustomed to constant stressors and so are less likely to recall them or recognise them as traumatic, or because they are already discriminated against because of the stigma attached to their neighbourhood and therefore withhold information to avoid negative judgments.

### 4.3 Implications for policy, practice, and future research

Our findings do not indicate that exposure to neighbourhood-level violence affects the way in which psychosis manifests. However, they do suggest that people living in high-violence areas may experience more coercion within mental health services than those from low-crime areas. This is not explained by an increased propensity for violence by people experiencing psychosis from these neighbourhoods; indeed our evidence suggests that they may be slightly less likely than those living in safer areas to have come into contact with mental health services due to violent acts. If replicated, this represents an important inequality in the treatment that people from different neighbourhoods receive. Residents of urban neighbourhoods with high rates of crime—which are typically low-income neighbourhoods—report widespread discrimination based on their area of residence regardless of their personal involvement in crime, due to the association of these neighbourhoods with violent gangs (25, 28). We hypothesise that this may explain the disparity in treatment that they receive. Health workers are not immune to the same stigmatising beliefs and attitudes held in the wider society, which can lead to unfair assumptions about individuals from certain communities and discrimination towards these groups. Further research is necessary to establish whether discrimination is occurring within mental health services and to evaluate locally-appropriate interventions to reduce unnecessary coercion within the health system (e.g., drawing on the WHO’s QualityRights programme (46)).

There is also some evidence that the psychological effects of violent crime may not be confined to the areas where such incidents are most likely to occur, with fear of crime also prevalent for those least likely to be personally victimised (47, 48). This suggests that in countries with high crime rates such as Trinidad, even neighbourhoods with lower rates of violent crime might experience similar psychological effects to those in identified hotspots. Consistent with this, the latest available data from the World Values Survey (from 2014) ranked Trinidad & Tobago second lowest in the world in terms of trust, with only 3.2% of respondents agreeing that most people can be trusted (49). This generalised—rather than localised—effect of crime could potentially explain the lack of associations reported in the present study. National-level policies to effectively tackle gang violence are urgently needed, in addition to community-level initiatives.

Finally, we found some counter-intuitive results, indicating higher rates of delusions of persecution in lower-violence than high-violence areas. As mentioned in the limitations section, is it possible that these findings arose because of systematic under-reporting by those living in contexts of frequent violence, where stressors may be normalised and where participants may be more wary of disclosing experiences that reinforce stereotypes about residents of neighbourhoods that are stigmatised due to their association with crime. We should therefore interpret these findings with caution. It is theoretically possible that constant stressors lead to adaptation and resilience, although this runs counter to our previous finding of increased risk of psychosis in urban areas where the prevalence of violence is greatest (27). We speculate that the findings could alternatively be attributable to participants and researchers taking context into account when considering symptoms, and thus being less likely to label experiences as pathological when there is a real possibility that the person is indeed in danger (e.g., rating an experience of persecution as delusional requires there to be no rational basis for believing that the person is actually being persecuted). This raises important conceptual and methodological questions for future psychiatric research in contexts where the risk of violence is high and where people can be targeted based on arbitrary characteristics such as which side of a gang boundary they live on.

## 5 Conclusion

This study found no evidence of an association between local rates of violent crime and the presentation or course of psychosis. It found tentative evidence that people from high-violence neighbourhoods may be more likely to experience coercion within mental health services, despite being no more likely to have come into services due to committing violent or threatening acts. Future research should investigate the causes of these disparities in treatment and evaluate initiatives to reduce coercion and discrimination within health services.

## Data Availability

The data analyzed in this study is subject to the following licenses/restrictions: the INTREPID II dataset is available upon reasonable request from the Principal Investigators. Requests to access these datasets should be directed to Craig Morgan, craig.morgan@kcl.ac.uk.

## Footnotes

^1^ https://cso.gov.tt/subjects/population-and-vital-statistics/crime-statistics/
