# Supplementary material for: Associations between local rates of violence and experiences of psychosis in Trinidad
Source: Front Public Health. 2025 Jun 11;13:1570957. doi: 10.3389/fpubh.2025.1570957 (PMC12188610; doi:10.3389/fpubh.2025.1570957)
Supplement: Supplementary file 1 [file Supplementary_file_1.pdf]

Table 1. Missingness by variable

Variable	Missing data - cases, n (%)	Missing data - controls, n (%)
Neighbourhood	0 (0)	0 (0)
Gender	0 (0)	0 (0)
Age	0 (0)	0 (0)
Ethnicity	0 (0)	0 (0)
Diagnosis	0 (0)	-
Delusions of persecution (SCAN score)	2 (0.9)	-
Delusions of reference (SCAN score)	1 (0.5)	-
Symptom dimensions: General factor	1 (0.5)	-
Symptom dimensions: Delusions factor	1 (0.5)	-
Symptom dimensions: Hallucinations factor	1 (0.5)	-
Symptom dimensions: Negative factor	1 (0.5)	-
Symptom dimensions: Disorganised factor	1 (0.5)	-
Symptom dimensions: Manic factor	1 (0.5)	-
Symptom dimensions: Depressive factor	1 (0.5)	-
Overall symptom severity at baseline (GAF 1)	2 (0.9)	-
Overall disability at baseline (GAF 2)	2 (0.9)	-
Frequency of cannabis use (ASSIST item)	11 (5.2)	3 (1.4)
Cannabis use (ASSIST score)	11 (5.2)	3 (1.4)
Witnessed any traumatic event (HTQ)	0 (0)	0 (0)
Experienced any traumatic event (HTQ)	0 (0)	0 (0)
Living alone	3 (1.4)	0 (0)
Committed violent or hazardous act	0 (0)	-
Behaviour seen as threatening or grossly annoying	0 (0)	-
Assaulted someone physically	0 (0)	-
Caused damage to property	0 (0)	-
Felt they were being harmed or persecuted	0 (0)	-
Arrested	0 (0)	-
First contact for psychosis	5 (2.4)	-
Admitted to hospital	0 (0)	-
Involuntary admission	0 (0)	-
Ever restrained	2 (0.9)	-
Any remission	28 (13.2)	-
No longer receiving treatment	21 (9.9)	-
Course of illness	27 (12.7)	-
In episode at follow-up	16 (7.6)	-
Overall symptom severity at 2 year follow-up (GAF 1)	37 (17.5)	-
Overall disability at 2 year follow-up (GAF 2)	37 (17.5)	-

The following tables report the results of the complete case analyses and the analyses with imputed data, to provide sensitivity analyses.

Table 2. Baseline risk/protective factors and their association with neighbourhood violence (cases and controls) – complete case analysis

	Lower-violence neighbourhoods (<10 per 100,000 population), n (%)	High-violence neighbourhoods (>10 per 100,000 population), n (%)	Unadjusted odds ratio (95% confidence interval)	p-value
Problematic cannabis use (ASSIST score>4)	98 (33.5)	42 (35.9)	1.11 (0.71-1.75)	0.64
Frequent cannabis use (weekly or more)	67 (22.9)	31 (26.5)	1.22 (0.74-1.99)	0.44
% experienced any traumatic event (HTQ)	264 (87.4)	112 (91.8)	1.61 (0.78-3.35)	0.20
% witnessed any traumatic event (HTQ)	116 (38.4)	54 (44.3)	1.11 (0.83-1.95)	0.27
Living alone	37 (12.3)	20 (16.5)	1.41 (0.78-2.54)	0.26
Afro-Trinidadian ethnicity	152 (50.3)	71 (58.2)	1.37 (0.90-2.10)	0.14

Table 2. Baseline risk/protective factors and their association with neighbourhood violence (cases and controls) – with imputed data

	Lower-violence neighbourhoods (<10 per 100,000 population), n (%)	High-violence neighbourhoods (>10 per 100,000 population), n (%)	Unadjusted odds ratio (95% confidence interval)	p-value
Problematic cannabis use (ASSIST score>4)	98 (33.5)	42 (35.9)	1.09 (0.67-1.77)	0.73
Frequent cannabis use (weekly or more)	67 (22.9)	31 (26.5)	1.16 (0.69-1.95)	0.58
% experienced any traumatic event (HTQ)	264 (69.1)	112 (64.7)	1.61 (0.78-3.35)	0.20
% witnessed any traumatic event (HTQ)	116 (38.4)	54 (44.3)	1.11 (0.83-1.95)	0.27
Living alone	37 (12.3)	20 (16.5)	1.34 (0.70 – 2.56)	0.38
Afro-Trinidadian ethnicity	152 (50.3)	71 (58.2)	1.37 (0.90-2.10)	0.14

Table 3a. Baseline presentation and neighbourhood violence (cases only – categorical variables) – complete case analysis

	Lower-violence neighbourhoods (<10 per 100,000 population), n (%)	High-violence neighbourhoods (>10 per 100,000 population), n (%)	Adjusted odds ratio* (95% confidence interval)	p-value
Affective diagnosis (F30.2,F31.2,F31.5, F32.3,F33.3)	63 (40.9)	18 (31.0)	1.51 (0.74-3.10)	0.26
Delusions of persecution (SCAN>1)	86 (56.6)	24 (41.4)	0.50 (0.26 – 0.97)	0.04
Delusions of reference (SCAN>1)	94 (61.4)	28 (48.3)	0.57 (0.30 – 1.09)	0.09

*Controlling for age, gender, ethnicity, and cannabis use

Table 3a. Baseline presentation and neighbourhood violence (cases only – categorical variables) – with imputed data

	Lower-violence neighbourhoods (<10 per 100,000 population), n (%)/mean (95% CI)	High-violence neighbourhoods (>10 per 100,000 population), n (%)/mean (95% CI)	Adjusted odds ratio *(95% confidence interval)	p-value
Affective diagnosis (F30.2,F31.2,F31.5, F32.3,F33.3)	63 (40.9)	18 (31.0)	1.51 (0.74-3.10)	0.26
Delusions of persecution (SCAN>1)	93 (61.1)	28 (48.3)	0.52 (0.28-0.99)	0.05
Delusions of reference (SCAN>1)	45 (29.8)	17 (29.8)	0.57 (0.31 1.08)	0.09

*Controlling for age, gender, ethnicity, and cannabis use

Table 3b. Baseline presentation and neighbourhood violence (cases only – continuous variables) – complete case analysis

	Lower-violence neighbourhoods (<10 per 100,000 population), mean (95% CI)	High-violence neighbourhoods (>10 per 100,000 population), mean (95% CI)	Adjusted beta coefficient* (95% confidence interval)	p-value
Symptom dimensions: General factor	0.56 (0.45-0.66)	0.50 (0.33-0.68)	-0.07 (-0.28 – 0.13)	0.47
Symptom dimensions: Delusions factor	0.12 (0.01-0.23)	0.05 (-0.13-0.23)	-0.09 (-0.31 – 0.12)	0.40

Symptom dimensions: Hallucinations factor	0.04 (-0.06-0.13)	-0.27 (0.45 - - 0.09)	-0.30 (-0.50– -0.10)	<0.01
Symptom dimensions: Negative factor	-0.04 (-0.15- 0.07)	-0.04 (-0.22-0.13)	-0.04 (-0.25– 0.17)	0.71
Symptom dimensions: Disorganised factor	-0.07 (-0.14-0.00)	-0.07 (-0.19-0.06)	-0.03 (-0.17– 0.11)	0.65
Symptom dimensions: Manic factor	0.28 (0.18-0.38)	0.25 (0.08-0.41)	-0.03 (-0.23 – 0.18)	0.81
Symptom dimensions: Depressive factor	0.51 (0.39-0.63)	0.36 (0.13-0.58)	-0.06 (-0.30 – 0.17)	0.61
Overall symptom severity at baseline (GAF 1)	62.1 (59.8–64.4)	61.5 (58.1–65.0)	0.78 (-5.42 – 6.98)	0.80
Overall disability at baseline (GAF 2)	60.9 (58.8–63.0)	59.3 (56.0–62.3)	-1.23 (-6.87– 4.42)	0.67

*Controlling for age, gender, ethnicity, and cannabis use

Table 3b. Baseline presentation and neighbourhood violence (cases only – continuous variables) – with imputed data

	**Lower-violence neighbourhoods (<10 per 100,000 population), mean (95% CI)**	**High-violence neighbourhoods (>10 per 100,000 population), mean (95% CI)**	**Adjusted beta coefficient* (95% confidence interval)**	**p-value**
Symptom dimensions: General factor	0.56 (0.45-0.66)	0.50 (0.33-0.68)	-0.03 (-0.23 –0.17)	0.76
Symptom dimensions: Delusions factor	0.12 (0.01-0.23)	0.05 (-0.13-0.23)	-0.08 (0.29 – 0.13)	0.43
Symptom dimensions: Hallucinations factor	0.04 (-0.06-0.13)	-0.27 (0.45 - - 0.09)	-0.30 (-0.50 – -0.11)	<0.01
Symptom dimensions: Negative factor	-0.04 (-0.15- 0.07)	-0.04 (-0.22-0.13)	-0.02 (-0.22 – 0.19)	0.86
Symptom dimensions: Disorganised factor	-0.07 (-0.14-0.00)	-0.07 (-0.19-0.06)	-0.02 (-0.15 – 0.12)	0.81
Symptom dimensions: Manic factor	0.28 (0.18-0.38)	0.25 (0.08-0.41)	-0.02 (-0.22 – 0.17)	0.80
Symptom dimensions: Depressive factor	0.51 (0.39-0.63)	0.36 (0.13-0.58)	-0.09 (-0.32 – 0.14)	0.46
Overall symptom severity at baseline (GAF 1)	62.1 (59.8–64.4)	61.5 (58.1–65.0)	-0.40 (-6.49 – 5.69)	0.90
Overall disability at baseline (GAF 2)	60.9 (58.8–63.0)	59.3 (56.0–62.3)	-1.87 (-7.63 – 3.62)	0.50

*Controlling for age, gender, ethnicity, and cannabis use

Table 4. Contact with mental health and justice systems and neighbourhood violence (cases only) – complete case analysis

	Lower-violence neighbourhoods (<10 per 100,000 population), n (%)	High-violence neighbourhoods (>10 per 100,000 population), n (%)	Adjusted odds ratio* (95% confidence interval)	p-value
Committed violent or hazardous act	47 (30.5)	14 (24.1)	0.76 (0.37 – 1.58)	0.47
Behaviour seen as threatening or grossly annoying	78 (50.6)	24 (41.4)	0.68 (0.36 – 1.30)	0.25
Assaulted someone physically	64 (41.6)	17 (29.3)	0.55 (0.27 – 1.10)	0.09
Caused damage to property	61 (39.6)	18 (31.0)	0.61 (0.31 – 1.20)	0.15
Felt they were being harmed or persecuted	111 (72.1)	40 (69.0)	0.92 (0.47 – 1.80)	0.81
Arrested	43 (27.9)	17 (29.3)	0.82 (0.39 – 1.72)	0.60
First contact for psychosis was police	15 (9.9)	6 (10.7)	0.88 (0.31 – 2.53)	0.82
Admitted to hospital	97 (63.0)	35 (58.6)	0.80 (0.42 – 1.53)	0.50
Involuntary admission	72 (46.8)	29 (50.0)	1.15 (0.61 – 2.15)	0.67
Ever restrained in services	26 (17.1)	18 (31.0)	2.00 (0.98 – 4.09)	0.06

*Controlling for age, gender, ethnicity, and cannabis use

Table 4. Contact with mental health and justice systems and neighbourhood violence (cases only) – with imputed data

	Lower-violence neighbourhoods (<10 per 100,000 population), n (%)	High-violence neighbourhoods (>10 per 100,000 population), n (%)	Adjusted odds ratio* (95% confidence interval)	p-value
Committed violent or hazardous act	47 (30.5)	14 (24.1)	0.76 (0.37-1.58)	0.47
Behaviour seen as threatening	78 (50.6)	24 (41.4)	0.68 (0.36 - 1.30)	0.25

or grossly annoying				
Assaulted someone physically	64 (41.6)	17 (29.3)	0.55 (0.27-1.10)	0.09
Caused damage to property	61 (39.6)	18 (31.0)	0.61 (0.31-1.20)	0.15
Felt they were being harmed or persecuted	111 (72.1)	40 (69.0)	0.92 (0.47-1.80)	0.81
Arrested	43 (27.9)	17 (29.3)	0.82 (0.39-1.72)	0.60
First contact for psychosis was police	15 (9.9)	6 (10.7)	0.87 (0.31-2.49)	0.80
Admitted to hospital	97 (63.0)	35 (58.6)	0.80 (0.42-1.53)	0.50
Involuntary admission	72 (46.8)	29 (50.0)	1.15 (0.61 - 2.15)	0.67
Ever restrained in services	26 (17.1)	18 (31.0)	2.00 (0.98-4.09)	0.06

*Controlling for age, gender, ethnicity, and cannabis use

Table 5a. Course/outcomes and neighbourhood violence (cases only – categorical variables) – complete case analysis

	**Lower-violence neighbourhoods (<10 per 100,000 population), n (%)**	**High-violence neighbourhoods (>10 per 100,000 population), n (%)**	**Adjusted odds ratio* (95% confidence interval)**	**p-value**
Any remission	128 (91.4)	39 (88.6)	0.95 (0.27 – 3.28)	0.93
No longer receiving treatment	82 (57.8)	28 (57.1)	0.98 (0.48 – 1.98)	0.96
Continuous course	23 (16.3)	7 (15.6)	0.80 (0.29 – 2.22)	0.66
In episode at follow-up	40 (27.6)	17 (33.3)	1.23 (0.59 – 2.55)	0.59

*Controlling for age, gender, ethnicity, and cannabis use

Table 5a. Course/outcomes and neighbourhood violence (cases only – categorical variables) – with imputed data

	**Lower-violence neighbourhoods**	**High-violence neighbourhoods (>10 per 100,000**	**Adjusted odds ratio* (95% confidence interval)**	**p-value**

	(<10 per 100,000 population), n (%)	population), n (%)		
Any remission	128 (91.4)	39 (88.6)	0.50 (0.18-1.42)	0.20
No longer receiving treatment	82 (57.8)	28 (57.1)	0.92 (0.46-1.83)	0.81
Continuous course	23 (16.3)	7 (15.6)	1.25 (0.48-3.23)	0.65
In episode at follow-up	40 (27.6)	17 (33.3)	1.33 (0.67-2.63)	0.41

*Controlling for age, gender, ethnicity, and cannabis use

Table 5b. Course/outcomes and neighbourhood violence (cases only – continuous variables) – complete case analysis

	Lower-violence neighbourhoods (<10 per 100,000 population), n (%)	High-violence neighbourhoods (>10 per 100,000 population), n (%)	Adjusted beta coefficient* (95% confidence interval)	p-value
Overall symptom severity at 2 year follow-up (GAF 1)	58.9 (55.3-62.5)	56.3 (50.2-62.4)	-1.36 (-8.38 – 5.67)	0.70
Overall disability at 2 year follow-up (GAF 2)	58.3 (54.7-62.0)	56.7 (50.2-63.2)	-0.69 (-7.86 – 6.48)	0.85

*Controlling for age, gender, ethnicity, and cannabis use

Table 5b. Course/outcomes and neighbourhood violence (cases only – continuous variables) – with imputed data

	Lower-violence neighbourhoods (<10 per 100,000 population), mean (95% confidence interval)	High-violence neighbourhoods (>10 per 100,000 population), mean (95% confidence interval)	Adjusted beta coefficient* (95% confidence interval)	p-value
Overall symptom severity at 2 year follow-up (GAF 1)	58.9 (55.3-62.5)	56.3 (50.2-62.4)	-1.97 (-8.77 – 4.82)	0.57
Overall disability at 2 year follow-up (GAF 2)	58.3 (54.7-62.0)	56.7 (50.2-63.2)	-1.38 (-8.23 – 5.48)	0.69

*Controlling for age, gender, ethnicity, and cannabis use
